# Raptor-Assisted Retrieval of a Radiolucent Perforating Intracardiac Foreign Body

**DOI:** 10.1016/j.jscai.2026.105341

**Published:** 2026-04-30

**Authors:** James F. Howick V, Guy Robinson, Christoff van Niekerk, Prajwal Reddy, Peter Pollak

**Affiliations:** a Department of Cardiovascular Disease, Mayo Clinic, Jacksonville, Florida; b Department of Internal Medicine, Mayo Clinic, Jacksonville, Florida

**Keywords:** hybrid catheter-based extraction, intracardiac echocardiography, intracardiac foreign body

Intracardiac foreign bodies are uncommon and may cause perforation, valvular injury, arrhythmias, or pericardial complications. Retrieval can be challenging when objects are long, fixed, involve the valvular apparatus, or traverse multiple chambers. Although open cardiac surgical removal is commonly required, percutaneous retrieval may be an option in carefully selected cases. However, optimal strategies for percutaneous remain ill-defined and typically rely on fluoroscopic visualization alone.

A 46-year-old woman presented 1 month after a Wolff-Parkinson-White ablation complicated by embolization and reported snare retrieval of a micropuncture dilator. Chest x-ray was unrevealing, yet cardiac computed tomography revealed a 10-cm linear fragment extending from the right atrium across the tricuspid valve into the right ventricle, with one end perforating the right atrial appendage and entering the pericardial space (Figure 1A). Transthoracic echocardiography confirmed a linear echogenic structure crossing the tricuspid valve with mild tricuspid regurgitation and a small pericardial effusion (Figure 1B).

**Figure 1:**
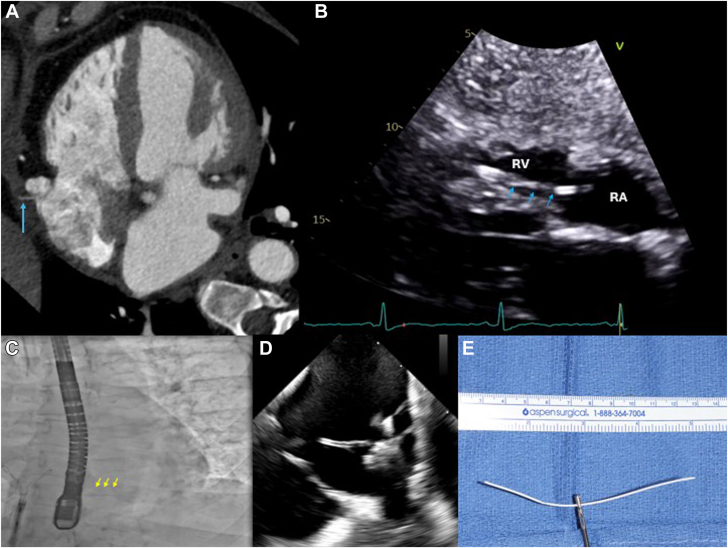
**Multimodality imaging and hybrid retrieval of a perforating intracardiac foreign body.** (**A**) Computed tomography demonstrating a 10-cm catheter fragment traversing the right atrium into the right ventricle, with proximal perforation into the pericardial space (arrow). (**B**) Transthoracic echocardiography showing the linear foreign body crossing the tricuspid valve (arrows). (**C**) Fluoroscopy during Needle’s Eye Snare attempt (arrows) demonstrating inability to directly visualize the radiolucent foreign body. (**D**) Intracardiac echocardiography demonstrating Raptor grasping of the fragment. (**E**) Retrieved intact 10-cm catheter fragment.

Given the fragment’s length, fixation, and transmural extension, the heart team elected hybrid operating room retrieval with surgical standby in anticipation of potential pericardial bleeding. Via femoral venous 16F access and a steerable sheath, retrieval was initially attempted using a Needle’s Eye Snare (Cook Medical), but secure engagement of the fragment could not be achieved because of the small caliber, flexibility, and radiolucency of the object (Figure 1C). The radiolucency of the object complicated retrieval as it was not visible on fluoroscopy and required transitioning to ultrasound guidance exclusively. In the absence of a free end suitable for snaring, a grasping strategy was pursued. Under combined intracardiac and transesophageal echocardiographic guidance, a Raptor endoscopic grasping device (STERIS Healthcare) was maneuvered to the fragment, achieving firm capture and allowing extraction of the intact 10-cm object (Figure 1D, E; Supplemental Video 1). Cross-plane imaging was used to ensure the forceps remained clear of the right atrial wall and tricuspid valve. No new pericardial effusion or valvular injury was observed.

The use of a Raptor grasping device has been previously described for larger, radiopaque objects; however, echocardiography-guided removal of a long, fixed, radiolucent, perforating intracardiac foreign body is novel. This case illustrates how hybrid percutaneous approaches, facilitated by multimodality imaging, may obviate the need for sternotomy in carefully selected patients.

## Declaration of competing interest

The authors declared no potential conflicts of interest with respect to the research, authorship, and/or publication of this article.

## Supplementary material

Supplemental Video 1 Intracardiac echocardiography-guided Raptor engagement of an intracardiac foreign body. Intracardiac echocardiography demonstrating secure engagement of an intracardiac foreign body with a Raptor grasping device.

## Footnotes

To access the supplementary material accompanying this article, visit the online version of the *Journal of the Society for Cardiovascular Angiography & Interventions* at 10.1016/j.jscai.2026.105341.

